# Longitudinal results from a dedicated chronic total coronary occlusions percutaneous coronary intervention program—a single-center experience

**DOI:** 10.1007/s12471-025-01988-7

**Published:** 2025-10-09

**Authors:** Yvemarie B. O. Somsen, Rohan S. Mansaram, Roel Hoek, Camila S. Pizarro Perez, Dicky K. Y. Yee, Stefan P. Schumacher, Wynand J. Stuijfzand, Jos W. R. Twisk, Bimmer E. P. M. Claessen, Niels J. Verouden, Ruben W. de Winter, Sebastiaan A. Kleijn, José P. Henriques, Alexander Nap, Paul Knaapen

**Affiliations:** 1 Departments of Cardiology, Amsterdam UMC, Vrije Universiteit Amsterdam, Amsterdam, The Netherlands; 2 Department of Cardiology, Utrecht Universitair Medisch Centrum, Utrecht, The Netherlands; 3 Epidemiology & Data Science, Amsterdam UMC, Vrije Universiteit Amsterdam, Amsterdam, The Netherlands; 4 Department of Cardiology Amsterdam UMC, Academic Medical Center, Amsterdam, The Netherlands

**Keywords:** Chronic total coronary occlusion, Percutaneous coronary intervention, Learning curve, Hybrid approach

## Abstract

**Objective:**

To provide insight into the longitudinal (> 10 years) results of a dedicated CTO PCI program in a single center.

**Background:**

Percutaneous coronary intervention (PCI) of chronic total occlusions (CTO) requires substantial operator experience. Dedicated CTO programs aim to increase technical success rates through sufficient case volume. However, longitudinal data beyond 10 years on such programs are scarce.

**Methods:**

We included 1185 patients who underwent CTO PCI in the Amsterdam University Medical Center between 2013 and 2024. Technical CTO PCI success was defined as thrombolysis in myocardial infarction flow grade 3 and < 30% residual stenosis. Procedural success was defined as technical success in the absence of in-hospital major adverse cardiovascular events. Multivariable logistic regression analyses were used to identify predictors for technical success.

**Results:**

Mean age was 66 ± 11 years; 81% were male. Overall technical CTO PCI success (92%) and procedural success (87%) rates were high and consistent. We observed temporal changes in wire crossing time (31 [7–56] to 23 [5–67] minutes), contrast volume (360 ± 160 to 210 ± 101 mL), and procedural time (90 [60–130] to 121 [80–165] minutes). Additionally, MACE rate improved from 13% (in 2013–2015) to 7% (in 2021–2024). Age ≥ 65 years, prior CABG, three-vessel disease, and an intermediate to high J‑CTO score (≥ 2) predicted technical failure.

**Conclusions:**

This study reports the longitudinal (> 10 years) results of a dedicated CTO PCI program, which confirms that high technical CTO PCI and procedural success rates can be achieved by a single center.

**Supplementary Information:**

The online version of this article (10.1007/s12471-025-01988-7) contains supplementary material, which is available to authorized users.

## What’s new?

- This longitudinal study evaluated technical and procedural trends in CTO PCI over a 10-year period within a high-volume, dedicated CTO program.
- Procedural efficiency improved over time, as reflected by reduced wire crossing time and contrast volume, despite stable patient and lesion complexity.
- Independent predictors of technical failure included age ≥ 65 years, mildly impaired LVEF, prior CABG, three-vessel disease, and a Japanese CTO score ≥ 2.

## Introduction

The treatment of chronic total occlusions (CTO) poses a major challenge in interventional cardiology [1]. Up to 20% of patients undergoing invasive coronary angiography (ICA) are found to have a CTO [2], yet it is estimated that percutaneous coronary intervention (PCI) of these lesions is performed in less than 5% of cases in contemporary interventional practice [3]. Prior to the implementation of the hybrid algorithm, the success rate of CTO PCI was historically low [4, 5]. The hybrid algorithm allows for a rapid exchange between four crossing strategies: antegrade wire escalation (AWE), antegrade dissection and re-entry (ADR), retrograde wire escalation (RWE), and retrograde dissection and re-entry (RDR) [6, 7]. In addition to the systemization of CTO PCI, the successful revascularization of CTOs relies on numerous factors, including substantial operator experience, proctoring, and careful patient selection [1, 8–10]. This principle was adopted by two dedicated CTO interventionalists (PK/AN) at Amsterdam University Medical Center and became the foundation of the CTO program. Initiated in 2013, this program aimed to achieve high technical CTO PCI success rates (∼ 90%) in combination with acceptable (≈ 3%) in-hospital major adverse cardiovascular event (MACE) rates. The present study reports the longitudinal results of a dedicated CTO PCI program in a single center over a period of more than 10 years. Moreover, we aimed to identify predictors for technical CTO PCI success.

## Methods

### Study population

This study prospectively recruited patients from a dedicated CTO PCI center (Amsterdam University Medical Center, the Netherlands, locations VU Medical Center and Academic Medical Center) between December 2013 and August 2024. All consecutive patients who were referred to the local Heart Team were screened for inclusion in this registry. The local Heart Team carried the final responsibility for selecting patients for CTO PCI, which was based on the assessment of coronary anatomy, ischemia and/or viability testing, and the presence of cardiac symptoms (angina and/or dyspnea on exertion). Referral for ischemia or viability testing was left to the discretion of the treating cardiologist. All study data were captured and managed using an online Electronic Data Capture system (Castor Electronic Data Capture, Amsterdam, The Netherlands). The present study was approved by the local Medical Ethics Committee and complied with the Declaration of Helsinki. All participants provided written informed consent.

### Angiographic and procedural characteristics

Angiographic and procedural characteristics were reviewed by two expert CTO observers (YS/SP) using ICA images acquired by a monoplane cardiovascular X‑ray system (Allura Xper FD 10/10, Philips Healthcare, Best, The Netherlands). Between 2013–2019, a CTO was defined as a luminal occlusion of a coronary artery with a documented or estimated duration of at least 3 months and no or minimal contrast penetration through the lesion (Thrombolysis in Myocardial Infarction [TIMI] flow grade 0–1) [11]. The updated description of a CTO was introduced in 2020 and defined a CTO as the complete absence of antegrade flow through the CTO body with a documented or presumed duration of ≥ 3 months [12]. All CTO lesions were evaluated using the Japanese CTO (J-CTO) score [13]. The Rentrop classification and Werner grade were applied to describe collateral morphology [14, 15]. Diseased vessels were defined as those with ≥ 70% visual stenosis in a native, non-bypassed coronary artery or ≥ 50% stenosis in a bypass graft; the number of diseased vessels was recorded accordingly. All CTO PCI procedures were performed by two dedicated operators (AN and PK), in adherence with the hybrid algorithm [16]. The successful CTO crossing strategy was defined as the final crossing technique (AWE, ADR, RWE, or RDR) used to cross the CTO lesion. The CTO and procedural characteristics of multiple CTOs, treated either consecutively or in a staged PCI, were not separately captured in our registry. As such, we considered the CTO vessel treated first as the target vessel. In case of a previous investment procedure (involving subintimal plaque modification during the initial CTO PCI attempt to prepare the target lesion for a subsequent staged procedure after 6–8 weeks), we considered the staged PCI to be the index procedure. All other periprocedural data were manually extracted from the electronic patient file. Same-day discharge, defined as no overnight hospital stay following CTO PCI, was pursued in all patients.

### Study aim and outcomes

This study aimed to report the longitudinal results of the introduction of a dedicated CTO PCI program in a single center over a period of more than 10 years. Furthermore, we sought to identify predictors for technical CTO PCI success. Technical CTO PCI success was defined as the combination of TIMI flow grade 3 and < 30% residual stenosis [17]. Secondary outcomes included procedural success, which was described as technical success in the absence of in-hospital MACE. In-hospital MACE was defined as a composite endpoint and included: all-cause death, non-fatal myocardial infarction (MI), emergency revascularization of the target vessel with either PCI or coronary artery bypass grafting (CABG) surgery, tamponade requiring pericardiocentesis or surgery, stroke, and contrast-induced nephropathy. The definition of non-fatal MI followed the Fourth Universal Definition of Myocardial Infarction [18]. If MI was suspected, cardiac biomarkers were collected. Access-site bleeding with a Bleeding Academic Research Consortium (BARC) score of ≥ 2 was defined as clinically relevant. Vascular access site complications included the occurrence of: acute arterial thrombosis, arterial perforation, arterial dissection, pseudoaneurysm, arteriovenous fistula, and retroperitoneal hematoma. Lastly, we performed Kaplan-Meier survival analysis to investigate the impact of successful versus failed CTO PCI on long-term mortality.

### Statistical analysis

Continuous variables with a normal distribution are presented as mean ± standard deviation; non-normally distributed data are presented as median [interquartile range]. For categorical variables, we used frequencies with percentages. Baseline, angiographic, and procedural characteristics are presented across 4 time periods: [a] 2013–2015, [b] 2016–2018, [c] 2019–2021, and [d] 2022–2024. The rationale for this distribution is to provide insight into the impact of a higher caseload. Survival curves were generated using the Kaplan-Meier method. The statistical difference between successful and failed CTO PCI was evaluated using the log-rank test; the univariable hazard ratio with 95% confidence interval was estimated using Cox proportional hazards regression. Furthermore, we performed univariable logistic regression analysis with a *p*-value threshold of < 0.10 to identify relevant predictors for technical CTO PCI success for each period. The following variables were considered clinically relevant, and separately entered into the model: age (< 64 or ≥ 65), body mass index (BMI, continuous), female sex, left ventricular ejection fraction (normal (> 55), mildly reduced (40–54), and moderate to severely reduced (< 39)), hypertension, hypercholesterolemia, diabetes mellitus, peripheral artery disease, prior MI, prior PCI, prior CABG, number of diseased vessels, CTO target vessel (right coronary artery, left anterior descending artery, right circumflex artery), CTO lesion characteristics (blunt cap, calcification, bending > 45 degrees, occlusion length ≥ 20 mm, re-try lesion, in-stent CTO lesion), final J‑CTO score (0–1, 2, ≥ 3), and period ([a] 2013–2015, [b] 2016–2018, [c] 2019–2021, and [d] 2022–2024). Each period reflected the cumulative case volume, serving as an indirect measure of operator experience and its impact on technical CTO PCI success. All variables that reached the threshold were subsequently entered into a multivariable logistic regression model, in which a *p*-value of < 0.05 was considered statistically significant. Dummy variables were created for categorical data, wherein the first category of each variable type was used as a reference. Statistical analyses were performed using SPSS software (IBM SPSS Statistics 28.0, Chicago, IL).

## Results

### Study population

**Fig. 1:**
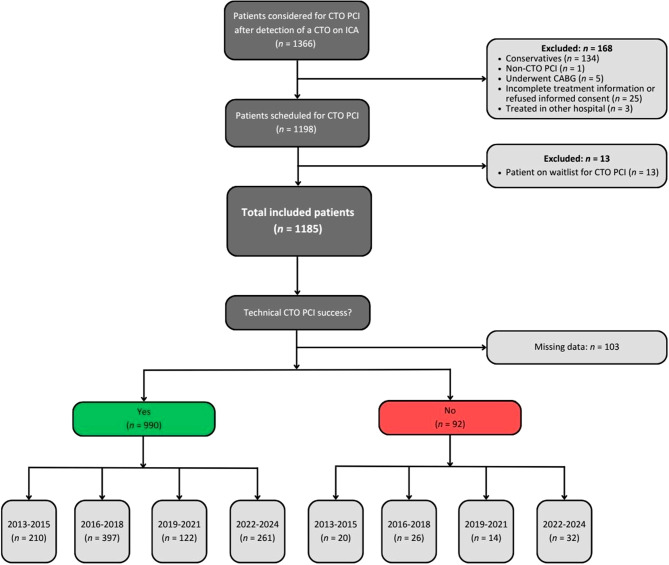
Study population flowchart. Flowchart of study subject inclusion. Technical CTO PCI success was defined as thrombolysis in myocardial infarction flow grade 3 and < 30% residual stenosis. Abbreviations: *CTO* chronic total coronary occlusion, *PCI* percutaneous coronary intervention, *ICA* invasive coronary angiography, *FU* follow-up

A total of 1366 patients were considered for CTO PCI after detection of a CTO on ICA (Fig. 1). Of this cohort, 181 patients were excluded due to: conservative treatment (*n* = 134), non-CTO PCI performed (*n* = 1), revascularization through CABG (*n* = 5), incomplete treatment information or no written informed consent (*n* = 25), treatment performed elsewhere (*n* = 3), or patient on the waitlist for CTO PCI (*n* = 13). The remaining 1185 patients were stratified according to the four predefined periods. The baseline characteristics are depicted in the Electronic Supplemental Material (ESM) Tab. S1. Age, BMI, and sex distribution were consistent across all time intervals. Hypertension, hypercholesterolemia, and family history of coronary artery disease demonstrated a higher prevalence in time period [c] and [d], whereas renal insufficiency showed a lower prevalence in time period [c] and [d]. The majority of patients in this cohort had a cardiac history of prior MI (54%) and prior PCI (60%).

### Angiographic and procedural characteristics

**Fig. 2:**
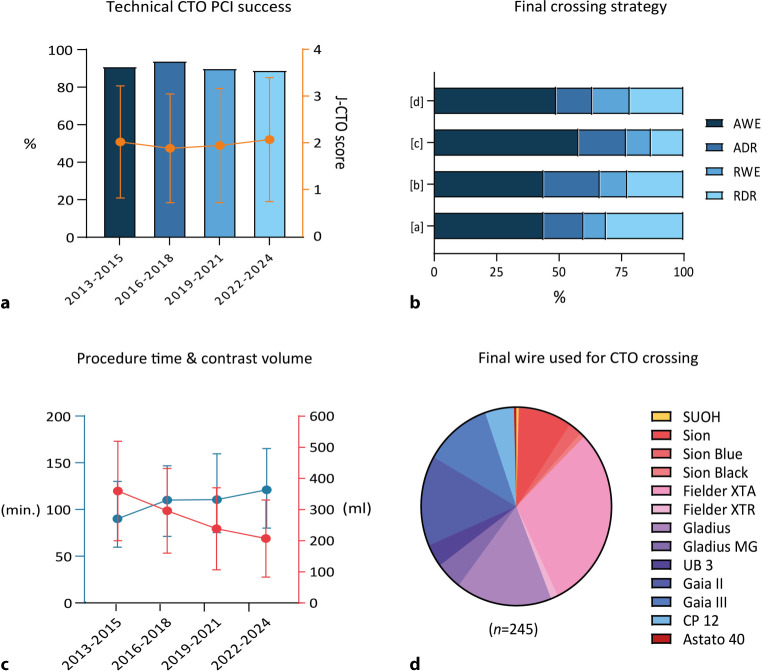
Procedural outcomes. **a** Technical CTO PCI success rates are constant, with an average Japanese CTO score (orange line) of 2 ± 1 across all time periods. **b** The most frequent final crossing strategy was AWE, followed by RDR. In time period (**c**), ADR was the second most used crossing strategy. **c** Total procedure time (blue line) showed a temporal increase, whereas total contrast volume (red line) decreased over time. **d** For 245 patients (21% of the total cohort), the final wire used to cross the CTO lesion was captured in the electronic patient dossier. The most frequently used wires were the Fielder XTA (30%), Gladius (16%), and Gaia II (15%), respectively

Angiographic characteristics are illustrated in Tab. 1. The majority of patients presented with single-vessel disease (63%), and the CTO was most commonly located in the RCA (60%). Of all CTO lesion characteristics, calcification (58%) and an occlusion length of ≥ 20 mm (47%) were most often reported. Re-try lesions and in-stent occlusions showed a gradual increase over time, and were highest in period [d] (20 and 17%, respectively). Rentrop classification 3 (81%) and Werner grade 2 (60%) were most often reported. Procedural characteristics are documented in Tab. 2. A radial-femoral access set-up was most commonly applied in the total cohort (69%), with the majority of patients (98%) receiving at least one large-bore (≥ 7 French) catheter. Among the 192 cases in which ADR was used, the specific re-entry technique was documented in 155 (81%) patients. In this subgroup, STAR (subintimal tracking and re-entry) was the most commonly employed technique (34%), followed by LAST (limited antegrade subintimal tracking) and use of the Stingray device (both 25%). Direct wire puncture from the subintimal space accounted for 11%, and the CrossBoss catheter was used in 5%. Across all time periods, AWE was most frequently noted as the final CTO crossing strategy (47%), followed by RDR (23%). Dual guiding catheter injection was applied in the vast majority of patients (91%). Total procedure time and radiation DAP demonstrated an increase over time, whereas wire crossing time and contrast volume gradually decreased (Fig. 2c). Finally, stent length was highest in time period [b] (91 mm ± 37) and lowest in time period [d] (67 ± 37).

**Table 1:** Angiographic characteristics

	Total cohort (*n* = 1185)	2013–2015 (*n* = 268)	2016–2018 (*n* = 483)	2019–2021 (*n* = 140)	2022–2024 (*n* = 294)
*CTO target vessel*					
RCA	705 (60)	175 (65)	289 (60)	81 (58)	160 (54)
LAD	296 (25)	59 (22)	108 (22)	41 (30)	88 (30)
LCX	173 (15)	34 (13)	83 (17)	17 (12)	39 (13)
LM	6 (1)	0 (0)	3 (1)	0 (0)	3 (1)
*Number of angiographically diseased vessels*					
1	681 (63)	179 (67)	311 (64)	82 (59)	109 (55)
2	287 (26)	74 (28)	129 (27)	34 (25)	50 (25)
3	119 (11)	15 (6)	43 (9)	23 (17)	38 (19)
*CTO lesion characteristics*					
Blunt cap*	338 (35)	83 (33)	127 (31)	52 (39)	76 (41)
Calcification	614 (58)	164 (61)	265 (55)	74 (54)	111 (59)
Bending > 45 degrees	417 (39)	110 (41)	189 (40)	46 (34)	72 (39)
Occlusion length ≥ 20 mm	503 (47)	124 (47)	219 (46)	66 (49)	94 (50)
Re-try lesion	136 (13)	40 (15)	41 (9)	18 (13)	37 (20)
In-stent occlusion	103 (10)	16 (6)	35 (7)	20 (15)	32 (17)
*Rentrop classification†*					
0–1	15 (3)	1 (1)	1 (1)	5 (5)	8 (4)
2	92 (17)	22 (15)	21 (16)	15 (16)	34 (19)
3	452 (81)	125 (85)	113 (84)	76 (79)	138 (77)
*Werner grade†*					
0	17 (2)	0 (0)	10 (2)	4 (3)	3 (2)
1	361 (36)	97 (38)	162 (38)	40 (32)	62 (35)
2	596 (60)	162 (63)	258 (60)	76 (60)	100 (57)
3	17 (2)	0 (0)	0 (0)	6 (5)	11 (6)
*J‑CTO score*					
0–1	396 (37)	92 (35)	186 (39)	52 (38)	66 (35)
2	321 (30)	85 (32)	151 (32)	37 (27)	48 (26)
≥ 3	350 (33)	90 (34)	140 (29)	47 (35)	73 (39)

Values are presented as *n* (%)

*RCA* right coronary artery; *LAD* left anterior descending artery; *LCX* left circumflex artery; *LM* left main; *J‑CTO* Japanese-CTO

*Cap morphology was available for 82%

†Rentrop classification and Werner grade were documented in 47% and 84% of cases

**Table 2:** Procedural characteristics

	Total cohort (*n* = 1185)	2013–2015 (*n* = 268)	2016–2018 (*n* = 483)	2019–2021 (*n* = 140)	2022–2024 (*n* = 294)
Arterial access set-up*					
Radial-femoral	750 (69)	160 (70)	285 (67)	90 (66)	215 (74)
Bifemoral	216 (20)	49 (21)	100 (24)	27 (20)	40 (14)
Biradial	10 (1)	3 (1)	3 (1)	2 (2)	2 (1)
Single access (femoral)	59 (6)	10 (4)	27 (6)	7 (5)	15 (5)
Single access (radial)	45 (4)	7 (3)	8 (2)	10 (7)	20 (7)
Large-bore (≥ 7 Fr) used	799 (98)	78 (94)	397 (99)	133 (99)	191 (98)
Number of treated vessels*					
1	693 (73)	159 (75)	269 (66)	83 (85)	182 (79)
2	203 (21)	44 (21)	108 (27)	9 (9)	42 (18)
3	51 (5)	9 (4)	29 (7)	6 (6)	7 (3)
Final CTO crossing strategy*					
AWE	480 (47)	94 (44)	179 (44)	76 (58)	131 (49)
ADR	192 (19)	34 (16)	93 (23)	25 (19)	40 (15)
RWE	114 (11)	19 (9)	43 (11)	13 (10)	39 (15)
RDR	234 (23)	65 (31)	94 (23)	17 (13)	58 (22)
*Procedural data†*					
Dual catheter injection	884 (91)	209 (93)	388 (92)	115 (86)	172 (90)
Wire crossing time (min)	31 [8–66]	31 [7–56]	35 [9–70]	24 [6–67]	23 [5–67]
Procedure time (min)	110 [72–152]	90 [60–130]	110 [72–147]	111 [75–160]	121 [80–165]
Fluoroscopy time (min)	40 [22–64]	35 [18–60]	40 [24–63]	42 [23–70]	41 [21–66]
Radiation DAP (Gycm^2^)	115 [60–185]	77 [44–135]	109 [62–172]	134 [71–207]	130 [59–211]
Skin dose (Gy)	1.4 [0.8–2.2]	1.2 [0.7–1.9]	1.4 [0.8–2.2]	1.6 [0.9–2.2]	1.4 [0.7–2.3]
Contrast volume (ml)	284 ± 143	360 ± 160	297 ± 136	239 ± 132	210 ± 101
Total stent length (mm)	83 ± 39	80 ± 38	91 ± 37	79 ± 42	67 ± 37
*Primary outcomes†*					
Technical success	990 (92)	210 (91)	397 (94)	122 (90)	261 (89)
Procedural success	942 (87)	200 (87)	378 (90)	117 (86)	247 (84)

Values are presented as mean ± SD, median [IQR] or *n* (%)

*AWE* antegrade wire escalation, *ADR* antegrade dissection and re-entry, *RWE* retrograde wire escalation, *RDR* retrograde dissection and re-entry, *DAP* dose area product

*Access site set-up, total number of vessels, and final CTO crossing strategy was captured in 91%, 80%, and 86% of cases, respectively. Whether a large-bore (≥ 7 French) catheter was used in at least one arterial access was known in 69% of patients

†Procedure time was documented in 78% of cases, fluoroscopy time in 71%, radiation DAP in 71%, skin dose in 65%, contrast volume in 62%

†Technical success and procedural success was noted in 91%

### Primary and secondary endpoints

Overall technical CTO PCI success was high (92%) and remained stable over time (Tab. 2). Similarly, procedural success was high (87%), and did not demonstrate a notable temporal change. In-hospital events are documented in the ESM Table S2. Local access site bleeding (13%) and perforation (11%) were most often reported complications resulting from CTO PCI. During the total study period, the MACE rate decreased from 13% (time period [a]) to 7% (time period [d]), respectively. Notwithstanding the temporal increase in bleeding events and vascular access complications, same-day discharge was achieved in 65%. Long-term mortality was lower in patients with successful versus failed CTO PCI (log-rank *p* < 0.01; hazard ratio 2.3, 95% CI: 1.6–3.5, *p* < 0.01), as shown in the Kaplan-Meier analysis (ESM Fig. S1).

### Identifying predictors for technical CTO PCI success

We performed multivariable logistic regression analyses to identify predictors for technical CTO PCI success (ESM Table S3). Age ≥ 65 years showed an association with decreased technical success rates (OR 0.54, 95% CI: 0.30–1.00), as well as a mildly impaired LVEF (OR 0.48 95% CI: 0.25–0.91), prior CABG (OR 0.56, 95% CI: 0.31–0.98), three-vessel disease (OR 0.41, 95% CI: 0.20–0.85), and an intermediate (2) and high (≥ 3) J‑CTO score (OR 0.31, 95% CI: 0.12–0.77; OR 0.25, 95% CI: 0.07–0.83). Although independent parameters of the J‑CTO score predicted technical failure in the univariable analyses (blunt cap, bending > 45 degrees, and occlusion length ≥ 20 mm), this finding did not persist in our multivariable model. Nevertheless, the combined impact of J‑CTO variables (resulting in a score of ≥ 2) predicted technical failure. Finally, we did not find a significant association between technical CTO PCI success and period.

## Discussion

This study presents the longitudinal results (> 10 years) of a dedicated CTO PCI program at a single high-volume center. Several key findings emerge from these data. First, we report high and consistent technical CTO PCI success and procedural success rates (92 and 87%, respectively, in the total cohort). Second, we observed temporal changes in important procedural characteristics: wire crossing time decreased from 31 [7–56] to 23 [5–67] minutes, contrast volume decreased from 360 ± 160 to 210 ± 101 milliliters, yet procedural time increased from 90 [60–130] to 121 [80–165] minutes. In addition, in-hospital MACE rate improved from 13% (in 2013–2015) to 7% (in 2021–2024). Third, the most frequently reported complications were local access site bleeding (13%) and perforation (11%), however, same-day discharge was feasible in the majority (65%) of patients. Finally, age ≥ 65 years, mildly impaired LVEF, prior CABG, three-vessel disease, and an intermediate to high J‑CTO score (≥ 2) predicted technical failure.

### The learning curve of CTO PCI

**Fig. 3:**
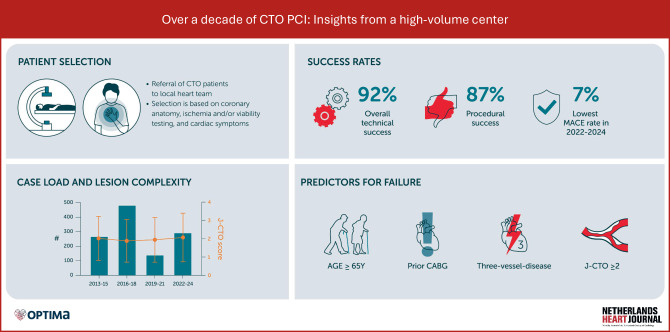
Infographic. We report the longitudinal (> 10 years) results following the introduction of a dedicated CTO program in Amsterdam University Medical Center. We found high and consistent technical CTO PCI success and procedural success rates (92 and 87% in the total cohort). The observed in-hospital MACE rate improved from 13% (in 2013–2015) to 7% (in 2021–2024). In this cohort, important predictors for technical failure were age ≥ 65 years, prior CABG, mildly impaired LVEF, prior CABG, three-vessel disease, and an intermediate to high J‑CTO score (≥ 2)

Experience is the bedrock of CTO PCI. To become a CTO operator, an interventionalist may rely on a combination of proctoring (either remote or through dedicated fellowships), a sufficient caseload (≥ 50 per annum [19]), and the reports of dedicated CTO programs. The latter provides valuable insight into the learning curve of CTO PCI and may offer a framework for the interventionalist who pursues their own CTO PCI program [20]. Although the longitudinal results of dedicated CTO PCI programs have been reported in abundance, the majority of study periods ranged from 12 months to 5 years [1, 10, 20–23]. To the best of our knowledge, the present study is the first to report the results of an extended (> 10 years) period in a high-volume Dutch CTO PCI center (Fig. 3). An extended study period may provide information on whether early gains in technical and procedural success rates are sustained over time, and whether complication rates decline accordingly. This is reflected in our data, as technical and procedural success rates remained high, while the in-hospital MACE rate showed a temporal decrease. Conversely, local access site bleeding showed a temporal increase within our study cohort—a notable finding that has also been reported previously by our research group [24]. In that report, we hypothesized that this increase may be attributable to the implementation of the CTO PCI fellowship program in 2017, as early-career interventionalists may be less familiar with the use of large-bore guiding catheters and dedicated vascular closure devices. An alternative explanation may lie in the introduction of an electronic data capture system in 2019, which could have enhanced the detection and reporting of bleeding events.

Similar to our study, prior reports focused on technical CTO PCI success and procedural success as their primary endpoints to demonstrate longitudinal results following the implementation of a CTO program [1, 25, 26]. Technical and procedural success rates aligned with these data [4, 21], despite a relatively high rate of prior CABG (22%), calcified lesions (58%), high (≥ 3) J‑CTO score (33%), and retrograde wiring techniques (34%) in our cohort—characteristics that may negatively impact these endpoints. The evidence of a so-called learning curve appears to be more prominent in other procedural parameters, such as the temporal decrease in wire crossing time, contrast volume, stent length, and in-hospital MACE rate. Although wire crossing time and contrast volume decreased, procedure time increased over the study period. This finding is likely multifactorial, and may be the result of screening for clinical trials (prolonging a procedure before exclusion criteria are confirmed), the start of the fellowship program, and a possible increase in the use of intracoronary imaging. Moreover, the observed decrease in stent length is relevant, given the established association between high stent burden and in-stent restenosis or target lesion failure [27, 28]. This may reflect a paradigm shift toward a more selective stenting strategy by operators over time, potentially influenced by a modest increase in the use of antegrade wire escalation techniques and a growing awareness of the long-term risks associated with extensive stenting. The observed MACE rate in our cohort exceeds the average percentage (≈ 3%) reported in various registry studies—a difference generally attributed to variations in lesion complexity [6]. Sapontis et al. [29] reported a major adverse cardiovascular and cerebrovascular event rate comparable to ours (7%) in the OPEN-CTO registry. Equal to our data, this registry included high rates of prior CABG (37%) and complex lesions (47% of participants had a J-CTO score > 2), and employed retrograde wiring techniques in ≈ 30% of cases. Prior CABG has been recognized as an important predictor for technical failure [4, 10], which our data confirmed. The presence of coexisting comorbidities, complex graft sequences, proximal cap ambiguity, high degrees of calcification, and a subsequent need for retrograde wiring techniques may all coexist in a single CABG patient [30]. As such, this patient category may benefit from careful referral, thorough preprocedural preparation (using non-invasive imaging), and proctoring by experienced operators early on in the CTO program.

### Limitations

Our study has several limitations. First, this is a registry-based study that only included patients who underwent CTO PCI. Second, we did not perform imputation on missing data, as these data are considered to be missing completely at random. Nevertheless, this could have influenced the identification of predictors for our primary outcome. Third, the COVID-19 pandemic resulted in a reduced sample size in the third period [c]. Fourth, we cannot provide insight into the impact of a single-operator or multi-operator approach on technical and procedural outcomes, as these data have not been systematically captured in the electronic patient dossier. Equally, the location of a prior attempt (external center or in-house) was not captured by our dataset. Fifth, the introduction of training programs for young interventionalists (i.e. fellowships) are an essential part of the CTO program in our center. The participation of such interventionalists in our hospital, albeit under strict supervision, may have impacted our results. Finally, cardiac biomarkers in case of a suspected MI were only routinely collected in period [a–b]. This could have led to an underestimation of the rate of non-fatal MI during period [c–d].

## Conclusions

The present study reported the longitudinal (> 10 years) results of the introduction of a dedicated CTO PCI program in one of the largest CTO centers in the Netherlands. Technical CTO PCI success rates and procedural success rates were high and consistent over time, emphasizing the successful introduction of the CTO program. The evidence of a learning curve could be found in several procedural characteristics, such as the temporal reduction in wire crossing time and contrast volume. Technical failure was driven by patient and case complexity, confirming that careful patient selection should remain one of the cornerstones of referral for CTO PCI.

## Supplementary Information

The Supplementary information includes a Kaplan-Meier survival analysis investigating the impact of successful versus failed CTO PCI on long-term mortality. Fig S1: Kaplan-Meier Survival curve

Tab S1: Baseline characteristics

Tab S2: In-hospital events

Tab S3: Multivariable logistic regression analyses to identify predictors for technical CTO PCI success
