# Supplementary material for: Longitudinal results from a dedicated chronic total coronary occlusions percutaneous coronary intervention program—a single-center experience
Source: Neth Heart J. 2025 Oct 9;33(11):361–9. doi: 10.1007/s12471-025-01988-7 (PMC12549449; doi:10.1007/s12471-025-01988-7)
Supplement: Supplementary file 1 — The Supplementary information includes a Kaplan-Meier survival analysis investigating the impact of successful versus failed CTO PCI on long-term mortality. Fig S1: Kaplan-Meier Survival curve [file 12471_2025_1988_MOESM1_ESM.docx]

**Longitudinal Results From a Dedicated Chronic Total Coronary Occlusions
Percutaneous Coronary Intervention Program***a single center experience*

Yvemarie B.O. Somsen, MD^1^, Rohan S. Mansaram, BSc^1^, Roel Hoek, MD^1^, Camila S. Pizarro Perez, MD^2^, Dicky K.Y. Yee, BSc^1^, Stefan P. Schumacher, MD^1^, Wynand J. Stuijfzand, MD, PhD^1^, Jos W.R. Twisk, PhD^3^, Bimmer E.P.M. Claessen, MD, PhD^4^, Niels J. Verouden, MD, PhD^1^, Ruben W. de Winter, MD^1^, Sebastiaan A. Kleijn, MD, PhD^1^, José P. Henriques, MD, PhD^4^, Alexander Nap, MD, PhD^1^, Paul Knaapen, MD, PhD^1^

Departments of ^1^Cardiology, ^3^Epidemiology & Data Science, Amsterdam UMC, Vrije Universiteit Amsterdam, Amsterdam, the Netherlands. ^2^Department of Cardiology, Utrecht Universitair Medisch Centrum, Utrecht, The Netherlands. Department of ^4^Cardiology Amsterdam UMC, Academic Medical Center, Amsterdam, the Netherlands.

**Conflict of interest:** Nothing to Disclose | **Sources of funding:** Dr. Paul Knaapen has received research grants from Cleerly Inc., and Heartflow Inc. Bimmer E.P.M. Claessen and José P. Henriques serve as editors for the Netherlands Heart Journal.

**Brief title:** Longitudinal Results of a CTO PCI Program

**Corresponding author:**

Paul Knaapen, MD, PhD

Professor of Cardiac Intervention & Imaging

Department of Cardiology Heart Center, Amsterdam UMC, location Vrije Universiteit Amsterdam

De Boelelaan 1117, 1081 HV Amsterdam, The Netherlands

Email: [p.knaapen@amsterdamumc.nl](mailto:p.knaapen@amsterdamumc.nl)

##
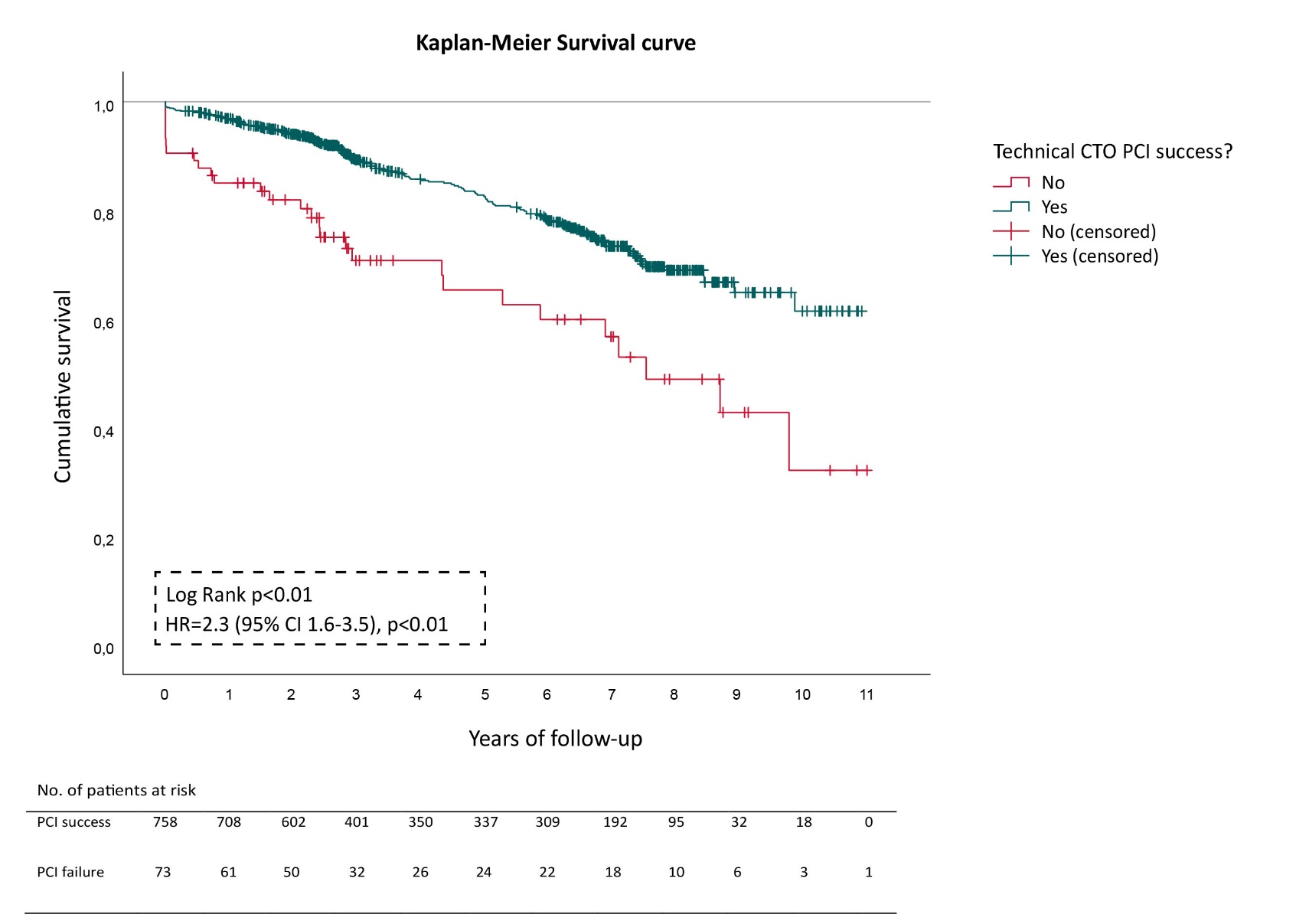
Figure 1. Long-term survival following successful versus failed CTO PCI

Figure 1 shows a Kaplan-Meier curve depicting long-term mortality in patients (n=833 of 1185) undergoing CTO PCI, stratified by successful versus failed CTO PCI.

## Supplemental Table S1. In-hospital events

|  | | | **Total cohort (*n* = 1185)** | **2013-2015**  **(*n* = 268)** | **2016-2018**  **(*n* = 483)** | **2019-2021**  **(*n* = 140)** | **2022-2024**  **(*n* = 294)** |
| --- | --- | --- | --- | --- | --- | --- | --- |
| *In-hospital events* | | |  |  |  |  |  |
|  | Perforation | | 122 (11) | 17 (7) | 56 (13) | 22 (16) | 27 (9) |
| *MACE* | | |  |  |  |  |  |
|  | MACE rate | | 130 (11) | 36 (13) | 59 (12) | 14 (10) | 21 (7) |
|  | Mortality | | 16 (2) | 2 (1) | 4 (1) | 3 (2) | 7 (2) |
|  | Non-fatal MI | | 61 (6) | 25 (11) | 29 (7) | 3 (2) | 4 (1) |
|  | Target vessel revascularization | |  |  |  |  |  |
|  |  | - *Emergency re-PCI* | 7 (1) | 1 (< 1) | 4 (1) | 0 (0) | 2 (1) |
|  |  | - *Emergency-CABG* | 0 (0) | 0 (0) | 0 (0) | 0 (0) | 0 (0) |
|  | Tamponade req. treatment | | 31 (3) | 6 (3) | 13 (3) | 4 (3) | 8 (3) |
|  | Stroke | | 4 (< 1) | 0 (0) | 4 (1) | 0 (0) | 0 (0) |
|  | Contrast induced nephropathy | | 11 (1) | 2 (1) | 5 (1) | 4 (3) | 0 (0) |
| *Vascular access complication* | | |  |  |  |  |  |
|  | VA complication occurred | | 56 (5) | 6 (3) | 15 (4) | 13 (10) | 22 (8) |
|  | Dissection | | 4 (< 1) | 1 (< 1) | 0 (0) | 2 (2) | 1 (< 1) |
|  | AV fistula | | 1 (< 1) | 0 (0) | 0 (0) | 1 (1) | 0 (0) |
|  | Thrombosis | | 1 (< 1) | 1 (1) | 0 (0) | 0 (0) | 0 (0) |
|  | Pseudoaneurysm | | 8 (1) | 1 (< 1) | 1 (< 1) | 1 (< 1) | 5 (2) |
|  | Hematoma | | 39 (4) | 1 (< 1) | 14 (3) | 9 (7) | 15 (5) |
| *Bleeding** | | |  |  |  |  |  |
|  | Bleeding event occurred | | 150 (14) | 13 (6) | 39 (9) | 30 (22) | 68 (23) |
|  | Local access site | | 137 (13) | 9 (4) | 35 (8) | 30 (22) | 63 (21) |
|  | Retroperitoneal | | 10 (1) | 3 (1) | 3 (1) | 0 (0) | 4 (1) |
|  | Gastro-intestinal | | 2 (< 1) | 1 (< 1) | 1 (< 1) | 0 (0) | 0 (0) |
|  | Intracerebral | | 0 (0) | 0 (0) | 0 (0) | 0 (0) | 0 (0) |
| *Admission to hospital* | | |  |  |  |  |  |
|  | Admission to hospital | | 377 (35) | 85 (37) | 163 (39) | 52 (39) | 77 (27) |
| Values are presented as n (%). *Data on bleeding events were available for 91%. VA: vascular access, other abbreviations as previously described. | | | | | | | |

## Supplemental Table S2. Predictors for technical CTO PCI success

| **Total cohort (*n*=1185)** | | | | |
| --- | --- | --- | --- | --- |
|  | **Univariable** | | **Multivariable** | |
|  | **OR (95% CI)** | ***p*-value** | **OR (95% CI)** | ***p*-value** |
| *Demographics* |  |  |  |  |
| Age ≥ 65 years | 0.48 (0.30-0.77) | < 0.01 | 0.54 (0.30-1.00) | 0.049 |
| BMI (continuous) | 0.97 (0.92-1.01) | 0.16 | - | - |
| Female sex | 0.92 (0.52-1.61) | 0.76 | - | - |
| *LVEF* |  |  |  |  |
| Mild, 40-54 | 0.64 (0.38-1.06) | 0.08 | 0.48 (0.25-0.91) | 0.03 |
| Moderate and severe, <39 | 0.78 (0.45-1.35) | 0.36 | - | - |
| *Cardiovascular risk factors* |  |  |  |  |
| Hypertension | 1.08 (0.70-1.67) | 0.72 | - | - |
| Hypercholesterolemia | 1.36 (0.88-2.10) | 0.16 | - | - |
| Diabetes mellitus | 0.92 (0.58-1.47) | 0.73 | - | - |
| Peripheral artery disease | 0.75 (0.44-1.27) | 0.28 | - | - |
| *Cardiac history* |  |  |  |  |
| Prior MI | 0.73 (0.48-1.13) | 0.16 | - | - |
| Prior PCI | 0.73 (0.47-1.14) | 0.17 | - | - |
| Prior CABG | 0.37 (0.23-0.57) | < 0.01 | 0.56 (0.31-0.98) | 0.04 |
| *# of diseased vessels* |  |  |  |  |
| 2 | 0.84 (0.49-1.45) | 0..53 | - | - |
| 3 | 0.43 (0.23-0.80) | < 0.01 | 0.41 (0.20-0.85) | 0.02 |
| *CTO target vessel* |  |  |  |  |
| LAD | 0.81 (0.49-1.33) | 0.40 | - | - |
| Cx | 0.77 (0.42-1.41) | 0.40 | - | - |
| *CTO lesion* |  |  |  |  |
| Blunt cap | 0.58 (0.35-0.95) | 0.03 | 0.90 (0.49-1.64) | 0.73 |
| Calcification | 0.73 (0.45-1.20) | 0.22 | - | - |
| Bending > 45 degrees | 0.49 (0.31-0.78) | < 0.01 | 0.80 (0.41-1.56) | 0.51 |
| Occlusion length ≥ 20 | 0.59 (0.37-0.94) | 0.03 | 1.05 (0.52-2.12) | 0.90 |
| Re-try lesion | 0.72 (0.39-1.35) | 0.31 | - | - |
| In-stent CTO | 0.81 (0.39-1.68) | 0.57 | - | - |
| *J-CTO score* |  |  |  |  |
| 2 | 0.39 (0.20-0.78) | < 0.01 | 0.31 (0.12-0.77) | 0.01 |
| ≥ 3 | 0.28 (0.14-0.52) | < 0.01 | 0.25 (0.07-0.83) | 0.02 |
| *Period* |  |  |  |  |
| [b] 2016-2018 | 1.45 (0.79-2.67) | 0.23 | - | - |
| [c] 2019-2021 | 0.83 (0.41-1.70) | 0.61 | - | - |
| [d] 2022-2024 | 0.78 (0.43-1.40) | 0.40 | - | - |
| For univariable analysis, *p*-value threshold of < 0.10 was applied. For multivariable analysis, statistical significance was set at *P*< 0.05. OR: odds ratio, other abbreviations as previously described. | | | | |
