# Supplementary material for: Longitudinal results from a dedicated chronic total coronary occlusions percutaneous coronary intervention program—a single-center experience
Source: Neth Heart J. 2025 Oct 9;33(11):361–9. doi: 10.1007/s12471-025-01988-7 (PMC12549449; doi:10.1007/s12471-025-01988-7)
Supplement: Supplementary file 2 — Tab S1: Baseline characteristics [file 12471_2025_1988_MOESM2_ESM.docx]

|  | | **Total cohort (*n* = 1185)** | **2013-2015**  **(*n* = 268)** | **2016-2018**  **(*n* = 483)** | **2019-2021**  **(*n* = 140)** | **2022-2024**  **(*n* = 294)** |
| --- | --- | --- | --- | --- | --- | --- |
| *Demographics* | | |  |  |  |  |
|  | Age, years | 66 ± 10 | 66 ± 11 | 66 ± 10 | 66 ± 10 | 66 ± 10 |
|  | BMI, kg · m^-2^ | 28 ± 7 | 28 ± 4 | 28 ± 5 | 28 ± 5 | 28 ± 4 |
|  | Male sex | 961 (81) | 225 (84) | 395 (82) | 109 (78) | 232 (79) |
| *LVEF, %* | | |  |  |  |  |
|  | Normal, ≥ 55 | 489 (43) | 77 (30) | 176 (38) | 74 (53) | 162 (56) |
|  | Mild, 40-54 | 339 (30) | 102 (40) | 144 (31) | 34 (24) | 59 (20) |
|  | Moderate and severe, ≤ 39 | 313 (27) | 77 (30) | 143 (31) | 30 (21) | 63 (22) |
| *Cardiovascular risk factors* | | |  |  |  |  |
|  | Hypertension | 667 (57) | 138 (52) | 261 (55) | 80 (58) | 188 (64) |
|  | Hypercholesterolemia | 592 (51) | 106 (40) | 203 (43) | 93 (67) | 190 (65) |
|  | Diabetes mellitus | 333 (28) | 72 (27) | 134 (28) | 44 (31) | 83 (28) |
|  | History of smoking | 639 (57) | 147 (56) | 259 (55) | 77 (62) | 156 (61) |
|  | Renal insufficiency | 194 (16) | 59 (22) | 104 (22) | 8 (6) | 23 (8) |
|  | Family history of CAD* | 405 (39) | 99 (38) | 147 (31) | 50 (48) | 109 (54) |
|  | Peripheral artery disease | 204 (17) | 45 (17) | 97 (20) | 12 (9) | 50 (17) |
| *Cardiac history* | | |  |  |  |  |
|  | Prior MI | 637 (54) | 169 (63) | 257 (53) | 68 (49) | 143 (49) |
|  | Prior PCI | 711 (60) | 176 (66) | 292 (61) | 84 (60) | 159 (54) |
|  | Prior CABG | 260 (22) | 44 (16) | 131 (27) | 29 (21) | 56 (19) |
| *Cardiac medication* | | |  |  |  |  |
|  | Salicylates | 1003 (85) | 240 (90) | 403 (83) | 116 (83) | 244 (83) |
|  | Dual antiplatelet therapy | 679 (57) | 151 (56) | 265 (55) | 82 (59) | 181 (62) |
|  | Oral anticoagulation | 153 (13) | 43 (16) | 91 (19) | 14 (10) | 5 (2) |
|  | β-blocker | 913 (77) | 215 (81) | 369 (76) | 106 (76) | 223 (76) |
|  | Calcium channel blocker | 336 (28) | 79 (30) | 134 (28) | 38 (27) | 85 (29) |
|  | Long-acting nitrate | 390 (33) | 64 (24) | 156 (32) | 49 (35) | 121 (41) |
|  | Statin | 989 (84) | 227 (85) | 411 (85) | 112 (80) | 239 (81) |
| *Clinical presentation†* | | |  |  |  |  |
|  | Anginal pain | 595 (60) | 99 (48) | 199 (53) | 87 (66) | 210 (75) |
|  | Dyspnea on exertion | 429 (44) | 57 (27) | 177 (47) | 56 (43) | 139 (50) |
| Values are presented as mean ± SD or n (%). *Family history was documented in 88% of cases; use of DOAC, proton pump inhibitor, and ACE inhibitor were documented in 34% of cases, respectively. **†**Clinical presentation was documented in 84% of cases. BMI: body mass index, LVEF: left ventricular ejection fraction, CAD: coronary artery disease, MI: myocardial infarction, PCI: percutaneous coronary intervention, CABG: coronary artery bypass grafting. | | | | | | |

## Table 1. Baseline characteristics
