# Supplementary material for: Longitudinal results from a dedicated chronic total coronary occlusions percutaneous coronary intervention program—a single-center experience
Source: Neth Heart J. 2025 Oct 9;33(11):361–9. doi: 10.1007/s12471-025-01988-7 (PMC12549449; doi:10.1007/s12471-025-01988-7)
Supplement: Supplementary file 3 — Tab S2: In-hospital events [file 12471_2025_1988_MOESM3_ESM.docx]

## Supplemental Table S1. In-hospital events

|  | | | **Total cohort (*n* = 1185)** | **2013-2015**  **(*n* = 268)** | **2016-2018**  **(*n* = 483)** | **2019-2021**  **(*n* = 140)** | **2022-2024**  **(*n* = 294)** |
| --- | --- | --- | --- | --- | --- | --- | --- |
| *In-hospital events* | | |  |  |  |  |  |
|  | Perforation | | 122 (11) | 17 (7) | 56 (13) | 22 (16) | 27 (9) |
| *MACE* | | |  |  |  |  |  |
|  | MACE rate | | 130 (11) | 36 (13) | 59 (12) | 14 (10) | 21 (7) |
|  | Mortality | | 16 (2) | 2 (1) | 4 (1) | 3 (2) | 7 (2) |
|  | Non-fatal MI | | 61 (6) | 25 (11) | 29 (7) | 3 (2) | 4 (1) |
|  | Target vessel revascularization | |  |  |  |  |  |
|  |  | - *Emergency re-PCI* | 7 (1) | 1 (< 1) | 4 (1) | 0 (0) | 2 (1) |
|  |  | - *Emergency-CABG* | 0 (0) | 0 (0) | 0 (0) | 0 (0) | 0 (0) |
|  | Tamponade req. treatment | | 31 (3) | 6 (3) | 13 (3) | 4 (3) | 8 (3) |
|  | Stroke | | 4 (< 1) | 0 (0) | 4 (1) | 0 (0) | 0 (0) |
|  | Contrast-induced nephropathy | | 11 (1) | 2 (1) | 5 (1) | 4 (3) | 0 (0) |
| *Vascular access complication* | | |  |  |  |  |  |
|  | VA complication occurred | | 56 (5) | 6 (3) | 15 (4) | 13 (10) | 22 (8) |
|  | Dissection | | 4 (< 1) | 1 (< 1) | 0 (0) | 2 (2) | 1 (< 1) |
|  | AV fistula | | 1 (< 1) | 0 (0) | 0 (0) | 1 (1) | 0 (0) |
|  | Thrombosis | | 1 (< 1) | 1 (1) | 0 (0) | 0 (0) | 0 (0) |
|  | Pseudoaneurysm | | 8 (1) | 1 (< 1) | 1 (< 1) | 1 (< 1) | 5 (2) |
|  | Hematoma | | 39 (4) | 1 (< 1) | 14 (3) | 9 (7) | 15 (5) |
| *Bleeding** | | |  |  |  |  |  |
|  | Bleeding event occurred | | 150 (14) | 13 (6) | 39 (9) | 30 (22) | 68 (23) |
|  | Local access site | | 137 (13) | 9 (4) | 35 (8) | 30 (22) | 63 (21) |
|  | Retroperitoneal | | 10 (1) | 3 (1) | 3 (1) | 0 (0) | 4 (1) |
|  | Gastro-intestinal | | 2 (< 1) | 1 (< 1) | 1 (< 1) | 0 (0) | 0 (0) |
|  | Intracerebral | | 0 (0) | 0 (0) | 0 (0) | 0 (0) | 0 (0) |
| *Admission to hospital* | | |  |  |  |  |  |
|  | Admission to hospital | | 377 (35) | 85 (37) | 163 (39) | 52 (39) | 77 (27) |
| Values are presented as n (%). *Data on bleeding events were available for 91%. VA: vascular access, other abbreviations as previously described. | | | | | | | |
