# Supplementary material for: Longitudinal results from a dedicated chronic total coronary occlusions percutaneous coronary intervention program—a single-center experience
Source: Neth Heart J. 2025 Oct 9;33(11):361–9. doi: 10.1007/s12471-025-01988-7 (PMC12549449; doi:10.1007/s12471-025-01988-7)
Supplement: Supplementary file 4 — Tab S3: Multivariable logistic regression analyses to identify predictors for technical CTO PCI success [file 12471_2025_1988_MOESM4_ESM.docx]

| **Total cohort (*n*=1185)** | | | | |
| --- | --- | --- | --- | --- |
|  | **Univariable** | | **Multivariable** | |
|  | **OR (95% CI)** | ***p*-value** | **OR (95% CI)** | ***p*-value** |
| *Demographics* |  |  |  |  |
| Age ≥ 65 years | 0.48 (0.30-0.77) | < 0.01 | 0.54 (0.30-1.00) | 0.049 |
| BMI (continuous) | 0.97 (0.92-1.01) | 0.16 | - | - |
| Female sex | 0.92 (0.52-1.61) | 0.76 | - | - |
| *LVEF* |  |  |  |  |
| Mild, 40-54 | 0.64 (0.38-1.06) | 0.08 | 0.48 (0.25-0.91) | 0.03 |
| Moderate and severe, <39 | 0.78 (0.45-1.35) | 0.36 | - | - |
| *Cardiovascular risk factors* |  |  |  |  |
| Hypertension | 1.08 (0.70-1.67) | 0.72 | - | - |
| Hypercholesterolemia | 1.36 (0.88-2.10) | 0.16 | - | - |
| Diabetes mellitus | 0.92 (0.58-1.47) | 0.73 | - | - |
| Peripheral artery disease | 0.75 (0.44-1.27) | 0.28 | - | - |
| *Cardiac history* |  |  |  |  |
| Prior MI | 0.73 (0.48-1.13) | 0.16 | - | - |
| Prior PCI | 0.73 (0.47-1.14) | 0.17 | - | - |
| Prior CABG | 0.37 (0.23-0.57) | < 0.01 | 0.56 (0.31-0.98) | 0.04 |
| *# of diseased vessels* |  |  |  |  |
| 2 | 0.84 (0.49-1.45) | 0..53 | - | - |
| 3 | 0.43 (0.23-0.80) | < 0.01 | 0.41 (0.20-0.85) | 0.02 |
| *CTO target vessel* |  |  |  |  |
| LAD | 0.81 (0.49-1.33) | 0.40 | - | - |
| Cx | 0.77 (0.42-1.41) | 0.40 | - | - |
| *CTO lesion* |  |  |  |  |
| Blunt cap | 0.58 (0.35-0.95) | 0.03 | 0.90 (0.49-1.64) | 0.73 |
| Calcification | 0.73 (0.45-1.20) | 0.22 | - | - |
| Bending > 45 degrees | 0.49 (0.31-0.78) | < 0.01 | 0.80 (0.41-1.56) | 0.51 |
| Occlusion length ≥ 20 | 0.59 (0.37-0.94) | 0.03 | 1.05 (0.52-2.12) | 0.90 |
| Re-try lesion | 0.72 (0.39-1.35) | 0.31 | - | - |
| In-stent CTO | 0.81 (0.39-1.68) | 0.57 | - | - |
| *J-CTO score* |  |  |  |  |
| 2 | 0.39 (0.20-0.78) | < 0.01 | 0.31 (0.12-0.77) | 0.01 |
| ≥ 3 | 0.28 (0.14-0.52) | < 0.01 | 0.25 (0.07-0.83) | 0.02 |
| *Period* |  |  |  |  |
| [b] 2016-2018 | 1.45 (0.79-2.67) | 0.23 | - | - |
| [c] 2019-2021 | 0.83 (0.41-1.70) | 0.61 | - | - |
| [d] 2022-2024 | 0.78 (0.43-1.40) | 0.40 | - | - |
| For univariable analysis, *p*-value threshold of < 0.10 was applied. For multivariable analysis, statistical significance was set at *P*< 0.05. OR: odds ratio, other abbreviations as previously described.  Tab S3: Multivariable logistic regression analyses to identify predictors for technical CTO PCI success | | | | |
